# Characterization of a Novel Variant of the Quinolone-Resistance Gene *qnrB* (*qnrB89*) Carried by a Multi-Drug Resistant *Citrobacter gillenii* Strain Isolated from Farmed Salmon in Chile

**DOI:** 10.3390/antibiotics10030236

**Published:** 2021-02-26

**Authors:** Christopher Concha, Claudio D. Miranda, Rodrigo Rojas, Felix A. Godoy, Jaime Romero

**Affiliations:** 1 Laboratorio de Patobiología Acuática, Departamento de Acuicultura, Universidad Católica del Norte, 1780000 Coquimbo, Chile; christopher.concha@ucn.cl (C.C.); rrojas@ucn.cl (R.R.); 2 Centro AquaPacífico, 1780000 Coquimbo, Chile; jromero@inta.uchile.cl; 3 Centro i~mar, Universidad de Los Lagos, 5480000 Puerto Montt, Chile; felix.godoy@ulagos.cl; 4 Laboratorio de Biotecnología, Instituto de Nutrición y Tecnología de los Alimentos (INTA), Universidad de Chile, Macul, 7810000 Santiago, Chile

**Keywords:** *qnrB*, *Citrobacter gillenii*, salmon farming, quinolones, antibiotics, aquaculture

## Abstract

The main objective of this study was to characterize using whole-genome sequencing analysis, a new variant of the *qnrB* gene (*qnrB89*) carried by a fluoroquinolone-susceptible bacterium isolated from mucus of farmed *Salmo salar* fingerling in Chile. *Citrobacter gillenii* FP75 was identified by using biochemical tests and 16S ribosomal gene analysis. Nucleotide and amino acid sequences of the *qnrB89* gene exhibited an identity to *qnrB* of 81.24% and 91.59%, respectively. The genetic environment of *qnrB89* was characterized by the upstream location of a sequence encoding for a protein containing a heavy metal-binding domain and a gene encoding for a N-acetylmuramoyl-L-alanine amidase protein, whereas downstream to *qnrB89* gene were detected the *csp* and *cspG* genes, encoding cold-shock proteins. The *qnrB89* gene was located on a large chromosomal contig of the FP75 genome and was not associated with the 10-kb plasmid and class 1 integron harbored by the FP75 strain. This study reports for the first time the carriage of a *qnrB* gene by the *C. gillenii* species, and its detection in a bacterial strain isolated from farmed salmon in Chile.

## 1. Introduction

In Chilean salmon farming, the low availability of efficient vaccines and the high prevalence of bacterial infections, such as Piscirickettsiosis caused by the intracellular pathogen *Piscirickettsia salmonis* [1,2,3], have stimulated the use of large amounts of antimicrobials, accounting for a total consumption of 334.1 tons during 2019 [4]. During the last few years the main antibiotics used in Chilean salmon farming were florfenicol, oxytetracycline and erythromycin, and to a much lesser extent the first-generation fluoroquinolone flumequine [4,5].

Fluoroquinolones belong to a class of synthetic antimicrobial agents with a broad spectrum of activity [6,7,8] and are widely used in the treatment of infections caused by Gram negative bacteria [9]. However, due to the extensive use of these antibiotics, there has been an important increase in the resistance to these drugs [10]. The most effective fluoroquinolone resistance mechanism is chromosomal mutations that alter the antibiotic target proteins, DNA gyrase or DNA topoisomerase IV and their drug-binding affinity, commonly conferring high levels of resistance in several bacterial species [11,12]. Furthermore, the acquisition of plasmid-acquired resistance genes producing either target protection proteins, drug modifying enzymes or drug efflux pumps are other mechanisms of fluoroquinolone resistance [12,13,14]. Functions of several plasmid-mediated quinolone resistance (sometimes labeled PMQR) genes include protection of quinolone target proteins, which are mediated by *qnr* genes, encoding proteins that protect DNA gyrase and topoisomerase IV against quinolones, thus preventing their activity [15,16].

It is well known that *qnr*-carrying bacteria exhibit only a low-level resistance to fluoroquinolones, but these bacteria can facilitate the emergence of resistant strains through the acquisition of other mechanisms of quinolone resistance, such as topoisomerase mutations and efflux action [8,17,18].

Currently, various studies report that prevalence of plasmid-mediated quinolone resistance, mainly encoded by the *qnr* genes is widespread, thus their occurrence among environmental bacteria is of high concern, but only a few studies addressing the occurrence of *qnr* genes among bacteria associated with aquaculture settings [19,20,21], or near fish farms [22] have been reported.

It must be noted that *qnr* gene carriage by bacteria isolated from reared fish in Chilean salmon farming has never been previously reported. Thus, this study adds important information on the detection of a *qnr* gene carried by bacteria of animal origin, and reporting for the first time the detection of a *qnr* gene in *Citrobacter gillenii* species.

It could be concluded that quinolone resistance genes could be detected among bacteria from reared salmon in Chile but not necessarily associated with plasmid elements with potential for horizontal transfer, thus not prompting an important risk to human public health.

The main aim of the study was to describe a new variant of the *qnrB* gene and its genomic environment carried by the multidrug resistant *C. gillenii* FP75 strain recovered from mucus of salmon reared in a Chilean salmon farm.

## 2. Results

### 2.1. Bacterial Identification

**Figure 1:**
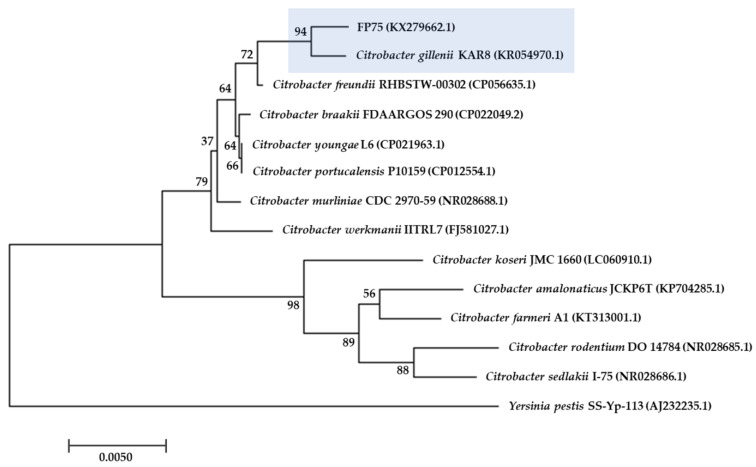
Phylogenetic tree based on the 16S rDNA gene sequence constructed by the neighbor-joining method. *Yersinia pestis* was used as an outgroup. Horizontal branch lengths are proportional to evolutionary divergences. Bootstrap values (%) appear next to the corresponding branch.

The bacterial strain FP75 was identified as *Citrobacter gillenii* by 16S ribosomal gene amplification (EMBL database accession number KX279662.1). The 16S rRNA gene sequence of strain FP75 was compared with all sequences currently available for members of the genus *Citrobacter* and related taxa. The results are presented as a phylogenetic dendrogram developed with the neighbor-joining method showing that FP75 strain is a member of the genus *Citrobacter*, where the most related species was *C. gillenii* (16S rRNA gene similarity 99.79%), as is depicted in Figure 1. In addition, identification of strain FP75 as *C. gillenii* was confirmed by specific biochemical characteristics, exhibiting important differences to the *C. freundii* species (Table 1).

### 2.2. Molecular Analysis

A qnr gene was detected in the genome of *C. gillenii* FP75 strain. The gene sequence was submitted to Dr. George Jacoby, curator of the qnr database, and responsible for the assignment of novel variants of qnr genes. This group confirmed the submitted gene as a unique sequence, naming this new variant of the qnrB gene, as qnrB89 (EMBL database accession number MT544491.1).

The new variant *qnrB89* showed a high identity to the sequences reported for the founding members of *qnrB* and *qnrE* genes included in the GenBank database (Table 2). Nucleotide sequence of *qnrB89* gene exhibited an 81.24% and 75.04% identity with the *qnrB* and *qnrE* genes, respectively, whereas at the amino acid sequence level, QnrB89 presents an identity of 91.59% and 83.64% with QnrB and QnrE, respectively (Table 2).

**Figure 2:**
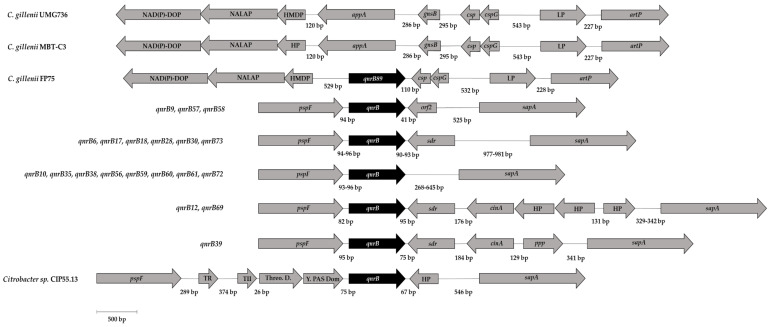
Comparison of the genetic environment of the gene qnrB89, Schematic representation of the genetic environment of qnrB89 gene carried by *Citrobacter gillenii* FP75 with several chromosomally located qnrB genes carried by various *Citrobacter* species as described by Ribeiro et al. [28]. Numbers between ORFs indicate the size of the intergenic region in base pairs (bp). The sequences used in the scheme were those previously considered by Ribeiro et al. [28], and include the GenBank accession numbers KP339254 (qnrB6), KP339255 (qnrB9), ADLG01000026.1 (qnrB9), KP339256 (qnrB10), CP007557 (qnrB12), KP339257 (qnrB17), KP339258 (qnrB18), ACDJ02000027.1 (qnrB18), JTBV01000001.1 (qnrB28), JMUJ01000007.1 (qnrB28), JAPA01000008.1 (qnrB30), JN173057 (qnrB35), JN173060 (qnrB38), NZ_AMPE01000004.1 (qnrB38), NZ_AKTT01000018.1 (qnrB38), NZ_AOUE01000004.1 (qnrB38), JTBJ01000001.1 (qnrB38), JAPB01000002.1 (qnrB38), ABWL02000005.1 (qnrB39), KP339259 (qnrB56), KP339260 (qnrB57), KP339261 (qnrB58), KP339262 (qnrB59), AB734055 (qnrB60), AB734053 (qnrB61), BBMW01000005.1 (qnrB69), KP339263 (qnrB72), KP339264 (qnrB73), and CDHL01000019, corresponding to a new qnrB from strain CIP 55-13T, as described by Ribeiro et al. [28]. In addition, similar locus found in two available *C. gillenii* genomes belonging to the not-carrying qnr genes strains *C. gillenii* UMG736 (SUQN00000000.1) and *C. gillenii* MBT-C3 (QVEK00000000.1), are included in the scheme.

The genetic environment of qnrB89 gene was characterized by the upstream location of a sequence encoding for a protein containing a heavy metal-binding domain (HMDP), a gene encoding a N-acetylmuramoyl-L-alanine amidase protein, which is a peptidoglycan hydrolase (NALAP) and a gene encoding for a NAD(P)-dependent oxidoreductase protein (NAD(P)-DOP), whereas downstream of the qnrB89 gene were detected two genes encoding cold shock proteins (csp and cspG), a gene that encodes a lipoprotein (LP), and the artP gene, encoding an arginine transport ATP-binding protein (Figure 2). As shown in Figure 2, the genetic environment of qnrB89 is not typical of those commonly described for the chromosomal located qnrB genes carried by various *Citrobacter* species.

When the FP75 genome was compared to the two publicly available *C. gilleni* genomes, corresponding to *C. gillenii* UMG736 (SUQN00000000.1) and *C. gillenii* MBT-C3 (QVEK00000000.1), composed of 24 and 37 contigs, respectively, no *qnr* gene was detected. However, it is interesting to note that in both genomes the *qnrB89* neighborhood was present, but instead of the *qnrB89* gene, gene *appA*, encoding for an AppA family phytase/histidine-type acid phosphatase protein and gene *gnsB*, encoding for an addiction module toxin, GnsA/GnsB family protein, were observed, located in contigs 1 and 9 of *C. gillenii* UMG736 and *C. gillenii* MBT-C3 genomes, respectively (Figure 2).

### 2.3. Microbial Susceptibility Profile and Minimum Inhibitory Concentrations (MICs)

MIC values of flumequine and enrofloxacin of *C. gillenii* FP75 and *Escherichia coli* UC238 carrying the *qnrB* gene are shown in Table 3. Assayed strains exhibited low MICs of flumequine and ciprofloxacin, observing slight differences. These results confirmed that carriage of the *qnrB89* gene is unable to confer resistance to quinolones in *C. gillenii* FP75 strain. Reference strain *E. coli* ATCC 25922, used for quality control exhibited a MIC value of flumequine and ciprofloxacin of 0.5 and 0.008 µg/mL, respectively, in agreement with the value recommended by CLSI [29].

Multidrug resistance of *C. gillenii* FP75 was demonstrated by performing a disk diffusion assay, showing resistance to the antibacterials amoxicillin, streptomycin, erythromycin, oxytetracycline, chloramphenicol, florfenicol, furazolidone, sulfisoxazole and trimethoprim and susceptibility to the antimicrobials cefotaxime, gentamicin, kanamycin, nalidixic acid, oxolinic acid, flumequine and ciprofloxacin (Table 3).

## 3. Discussion

The antimicrobials used in Chilean salmon farming are mainly florfenicol and oxytetracycline, accounting for the 90.8 and 99.8%, of used antibiotics during 2019 in freshwater and marine farms, respectively [4]. Thus, the use of oxolinic acid and flumequine, the unique quinolones authorized to be used in this industry is currently minimal [2,4,5].

Previous studies evidence *Citrobacter* as the origin of *qnrB* genes and suggest a divergent evolution of closely related *qnrB* genes [28,32]. *Citrobacter* are found in various clinical and environmental sources, including soil and water [33]. Furthermore, *Citrobacter* is currently considered an opportunistic pathogen in fish aquaculture, causing gastroenteritis of rainbow trout *Oncorhynchus mykiss* [34,35,36,37,38]. To date, a few studies have reported the isolation of *C. gillenii* from rainbow trout intestinal tract showing disease symptoms [24], and from the healthy fish intestine of rainbow trout and farmed grass carp (*Ctenopharyngodon idellus*) [39,40].

To the best of our knowledge, this is the first report of a *qnr* gene detected in the *C. gillenii* species, and prompts the necessity to investigate the carriage of *qnr* genes in farmed salmonid microbiota, and to provide a scientific basis of the prevalence of this opportunistic pathogen in farmed salmon to prevent potential secondary infections.

The higher similarity of nucleotide and amino acid sequence of *qnrB89* with the *qnrB* allele, when compared to the *qnrE* sequences, determined that this gene was classified as *qnrB89*. As was stated by Jacoby et al. [32], *qnrB* is the most common of the five *qnr* families and has the greatest number of allelic variants, being mostly detected in the *Citrobacter* genus with several of them located on the bacterial chromosome. Among the *qnrB* alleles, *qnrB89* variant exhibited the highest degree of amino acid (93.46%) sequence identity to the *qnrB12* sequence from *Citrobacter werkmanii* isolated from poultry [41].

However, the genetic environment of the *qnrB*89 gene is very different to those reported for most of the other *qnrB* alleles, which consistently include the *pspF* (encoding a phage shock protein) and *sapA* (encoding a protein involved in antimicrobial peptide resistance) genes upstream and downstream of the *qnrB* genes, respectively [28], which are absent in the *qnrB89* background. Furthermore, the *qnrB89* genetic background is also very different to those of the *qnrE1* gene, which is flanked by the genes *araJ* (encoding a arabinose efflux permease) and *ppk* (encoding a polyphosphate kinase) or *tnp* (encoding a transposase) [28,42]. Ribeiro et al. [28] characterized the genetic surroundings of various *qnrB* genes, observing eight different conserved genetic platforms for closely related *qnrB* genes carried by different *Citrobacter* species, demonstrating an association between the *qnrB* platforms carrying closely related *qnrB* genes and specific *Citrobacter* species. However, these genetic environments are highly different to that observed for the *qnrB89* gene of *C. gillenii* FP75. However, it should be noted that *C. gillenii* species was not included in the referenced study.

In previous studies, *qnrB* genes have been commonly associated with integrons in clinic isolates belonging to the *Citrobacter* genus [43]. Lee et al. [44] studied the genetic context surrounding chromosomal *qnrB62* gene carried by a *C. freundii* clinical isolate, observing an association with a complex class 1 integron. Furthermore, Ferreira et al. [45] isolated a clinical multiresistant *C. freundii* strain carrying a *qnrB* gene associated with a class 1 integron inserted in a large plasmid. In another study, several ciprofloxacin-resistant *C. freundii* recovered from wastewater treatment plants carried a *qnrB* gene as part of a complex integron [46]. Despite the previous finding that *C. gillenii* FP75 carries a class 1 integron [47], this study demonstrated that the harbored integron was not associated with the *qnrB89* gene, which is in agreement with other previous studies of aquaculture associated bacteria carrying *qnrB* genes [48,49].

Otherwise, unlike *qnrB89* gene, many reported *qnrB* alleles are associated with plasmid elements and are able to be horizontally transferred [50,51,52,53], but other *qnrB* variants have previously been described as non-transferable, such as the *qnrB12* variant carried by *Citrobacter werkmanii* and a *qnrB* variant carried by *Rhodococcus* sp., which were unable to be horizontally mobilized [48,54]. However, the carriage of transferable determinants conferring resistance to florfenicol and oxytetracycline of *C. gillenii* FP75, which are associated with the 10-kb plasmid [47], suggest that intensive use of these antibiotics in Chilean salmon farming will exert a selective pressure on this bacteria, promoting the co-selection and persistence of the detected *qnrB89* gene in the salmon mucus microbiota.

Despite the carriage of a variant of the *qnrB* gene by *C. gillenii* FP75, this strain is susceptible to quinolones. It is well known that resistance to quinolones is mainly due to chromosomal point mutations rather than being carried on any mobile genetic elements, but although *qnr* genes only confer low-level resistance to quinolones, these genes could favor the selection of additional chromosome-encoded quinolone resistance mechanisms. Furthermore, the study of *qnr* genes has an epidemiological relevance to advance a comprehensive understanding of the resistome associated with environmental settings, and to know their evolution and spread in these environments.

The chromosomal mutations exhibited by the *C. gillenii* FP75 strain leading to amino acid substitutions in the quinolone-resistance-determining regions of bacterial protein targets of quinolones were only a conserved change (Ser-83 to Thr) in the gyrase A protein, but not conferring resistance or low level susceptibility to quinolones, whereas the detected amino acid substitutions in GyrB (Leu-417 to His) and ParE (Ile-485 to Val) are located in protein regions not associated with changes in the susceptibility to quinolones.

In Chile in 2014 and 2015, the genes *qnrA*, *qnrB,* and *qnrS*, were described in strains isolated from uncontaminated sediments and sectors adjacent to a site affected by salmon farming [22,54]. However, this is the first report of a *qnr* gene detected in a bacterium directly associated with Chilean salmon farming, and most importantly, from farmed salmon mucus. The finding of this gene in a bacterial strain exhibiting susceptibility to quinolones suggest that incidence of *qnr* genes in these systems could be strongly underestimated when they are not associated with fluoroquinolone-resistant bacteria. Furthermore, these *qnr*-carrying bacteria are most frequently resistant to other antimicrobials, such as florfenicol and oxytetracycline, intensively used in Chilean aquaculture, thus favoring the prevalence of *qnr* genes in Chilean salmon farming, constituting a potential threat for salmon handlers and consumers, mostly considering that the chromosome of *Citrobacter* is the likely source of plasmid-mediated *qnrB*, as was suggested by Jacoby et al. [32].

Thus, the role of Chilean salmon farming as a potential reservoir of *qnr* genes must be elucidated, prompting the necessity of detecting *qnr* genes in a high number of representatives of reared salmon microbiota, including isolates exhibiting various levels of susceptibility to quinolones.

## 4. Materials and Methods

### 4.1. Bacterial Strain

The FP75 strain was recovered from mucus of reared salmon fingerling cultured in a freshwater farm located at the Puyehue Lake, in the South of Chile [47]. The purified strain stored at −85 °C in CryoBank^TM^ vials (Mast Diagnostica, Reinfeld, Germany), was grown in Trypticase soy agar (TSA, Oxoid, Hants, UK) at 30 °C for 24 h prior to use.

### 4.2. Bacterial Identification

The phenotypic tests Gram staining, cell morphology, oxidase production and oxidation/fermentation (O/F) of glucose were determined according to the procedures described in Buller [55]. In addition, biochemical properties, urease production, ornithine decarboxylation, fermentation of sucrose, utilization of acetate and malonate were determined to phenotypically differentiate the FP75 strain from the *Citrobacter freundii* species [56], using the procedures described by Barrow and Feltham [57].

Furthermore, FP75 strain was identified by bacterial 16S rRNA gene sequence analysis. DNA extraction and the amplification of the 16S ribosomal gene of the FP75 strain was performed as was previously described [47]. The sequence was edited and matched to the Ribosomal Database Project [58] to identify the bacterial isolate and deposited in the GenBank under accession number KX279662.1 as was previously reported [46]. The phylogenetic and molecular evolutionary analyses were conducted using MEGA version 7.0 [59].

### 4.3. Molecular Analysis of the qnrB89 Gene

Genomic DNA was extracted and purified using the commercial Wizard^®^ Genomic DNA Purification kit (Promega, Madison, WI, USA), following the indications of the supplier. The characterization of the *qnr* gene and its genetic environment was carried out through the complete sequencing of the bacterium’s genome. The whole genomic DNA was sequenced by Macrogen USA (Rockville, MD, USA). The analysis of the *qnr* gene sequence and its genetic environment was based on the contigs derived from genomic sequencing. This analysis was performed with the BioEdit 7.2.5 software [60] and subsequent comparison by BLAST computational analysis with the sequences described in the GenBank database. The identification and classification of the detected *qnr* gene was complemented with the group of experts led by Dr. George Jacoby.

### 4.4. Antimicrobial Resistance Pattern

The antimicrobial susceptibility to 16 antimicrobials of strains *C. gillenii* FP75, *E. coli* DH5α and transconjugant strain *E. coli* FP75T was determined using a disk diffusion test according to the Clinical and Laboratory Standards Institute (CLSI) guideline VET3-A [61] and previously described [62]. Briefly, bacterial suspensions in phosphate buffered saline at a turbidity corresponding to a 0.5 McFarland standard (bioMérieux, Marcy-l’Etoile, France) were streaked onto plates containing cation-adjusted Mueller–Hinton agar (CAMH, Difco Labs, NJ, USA) to which were added disks (Oxoid) containing the antibiotics amoxicillin (AML, 25 µg), cefotaxime (CTX, 30 μg), chloramphenicol (CM, 30 μg), florfenicol (FFC, 30 μg), streptomycin (S, 10 μg), gentamicin (CN, 10 μg), kanamycin (K, 30 μg), oxytetracycline (OT, 30 μg), erythromycin (E, 15 μg), nalidixic acid (NA, 30 μg), oxolinic acid (OA, 2 μg), flumequine (UB, 30 μg), ciprofloxacin (CIP, 5 μg), furazolidone (FR, 100 μg), sulfisoxazole (SFX, 300 μg) and trimethoprim (TMP, 5 μg). Plates were incubated at 28 °C for 24 h according to CLSI guidelines [61], and strains were considered resistant according to the criteria established by the CLSI [61,63]. As recommended by the CLSI guidelines [64], the reference strain *E. coli* ATCC 25922 was used as a quality control strain. All strains were re-examined to check the reproducibility of the assay.

### 4.5. Minimum Inhibitory Concentrations (MICs)

Minimum inhibitory concentrations (MICs) of flumequine and ciprofloxacin of *C. gillenii* FP75 and *E. coli* UC238 were determined by a microdilution method, as recommended by the CLSI guideline M07-A10 [65] and previously described [62]. Conical bottom microplates added with cation-adjusted Mueller–Hinton broth were inoculated with the antibiotic to obtain final series of two-fold concentrations in the range of 0.0625–128 µg/mL. Bacterial suspensions were prepared in sterile 0.85% saline and triplicate microplates were inoculated, delivering approximately 10^4^ colony-forming units per well, and incubated for 24 h at 28 °C. The reference strain *E. coli* ATCC 25922 was included as quality control, as was recommended [65]. All assays were performed twice to check the reproducibility of the assay.

A breakpoint of ≤1.0 µg/mL was used to consider susceptibility to ciprofloxacin as stated by the CLSI [30] for enteric bacteria. Considering that no MIC breakpoints for flumequine are currently stated, we categorized the isolates using as a reference the flumequine epidemiological cut-off (ECOFF) value stated by the European Committee on Antimicrobial Susceptibility Testing (EUCAST) [31] for *Escherichia coli* and *Salmonella* spp. (≤2.0 µg/mL for susceptible).

## 5. Conclusions

In conclusion, the results of this study demonstrated for the first time the carriage of a new *qnrB* variant (*qnrB89*) by a bacterial strain isolated from reared fish in Chilean salmon farming, and the detection of a *qnr* gene in the *Citrobacter gillenii* species. The detection of a *qnr* gene carried by a quinolone susceptible strain strongly suggests that farmed fish is an important reservoir of these genes but are significantly underestimated considering that *qnr* genes are almost exclusively investigated among quinolone-resistant bacteria. Furthermore, the uncommon genetic environment of the *qnrB89* gene, compared to other *qnrB* genes, and its non-association to integrons or plasmids suggest a most probable environmental origin, not related to a clinical source. This study shows important information on the characteristics of a *qnr* gene carried by quinolone susceptible bacteria from salmon farming, thus providing an important basis to advance the performing of genetic epidemiology studies on quinolone resistance genes in fish farm associated environments in Chile.

It can be concluded that quinolone resistance genes can be detected among bacteria from reared salmon in Chile but not necessarily associated with transferable elements, reducing their ability to be horizontally transferred, thus not significantly contributing to spread of these genes in these environments and not prompting an important risk to human public health.

## Figures and Tables

**Table 1:** Phenotypic differentiation between *Citrobacter gillenii* and *Citrobacter freundii*.

Species	Phenotypic Properties						Reference
	IND	URE	ODC	SUC	ACE	MAL	
FP75	+/delayed	−	−	−	−	+	
*C. gillenii*	+/delayed	−	−	−/+	−	+	[23,24]
*C. freundii*	+	+	+	+	+	−	[23,25]

IND: Indole production; URE: Urease; ODC: Ornithine decarboxylase; SUC: Sucrose fermentation; ACE: Acetate utilization; MAL: Malonate utilization; −: Negative; +: Positive; −/+: Negative or Positive.

**Table 2:** Similarity of nucleotide and amino acid sequences of *qnrB89* carried by *Citrobacter gillenii* FP75 with those of the alleles of the *qnr* gene.

Sequence	Percentage of Identity (%)							Reference
	*qnrA*	*qnrB*	*qnrC*	*qnrD*	*qnrE*	*qnrS*	*qnrVC*	
Nucleotide *	46.20	81.24	47.13	64.50	75.04	49.30	49.15	[26]
Amino acid **	40.19	91.59	42.52	65.42	83.64	40.65	42.52	[27]

*: *qnrA*, NG_050462.1; *qnrB*, NG_050469.1; *qnrC*, NG_048054.1; *qnrD*, NG_050541.1; *qnrE*, NG_054677.1; *qnrS*, NG_050543.1; *qnrVC*, NG_050551.1; **: QnrA, WP_012579084.1; QnrB, WP_014386481.1; QnrC, WP_032492368.1; QnrD, WP_012634451.1; QnrE, WP_061586512.1; QnrS, WP_001516695.1; QnrVC, WP_000415714.1.

**Table 3:** Antimicrobial resistance patterns and minimum inhibitory concentrations (MICs) of *Citrobacter gillenii* FP75 and *Escherichia coli* UC238.

Strain	MIC (µg/mL) of:		Antimicrobial Resistance to:
	FLQ	CIP	
*C. gillenii* FP75	0.25	0.015	AMO, STR, ERY, OXY, CHL, FLO, FR, SFX, TMP
*E. coli* UC238	0.5	0.25	ND

FLQ: Flumequine; CIP: Ciprofloxacin; AMO: Amoxicillin; STR: Streptomycin; ERY: Erythromycin; OXY: Oxytetracycline; CHL: Chloramphenicol; FLO: Florfenicol; FR: Furazolidone; SFX: Sulfisoxazole; TMP: Trimethoprim; ND: Not determined; Breakpoint for susceptibility to ciprofloxacin of ≤ 1.0 µg/mL was used as recommended by CLSI [30]; ECOFF value for susceptibility to flumequine of ≤ 2.0 µg/mL was used as recommended by EUCAST [31].

## Data Availability

The whole-genome sequence of FP75 strain has been deposited at DDBJ/ENA/GenBank under the accession number JAFDOE000000000 (BioProjectPRJNA699318; BioSample SAMN17773723).
